# Utility of Survival Motor Neuron ELISA for Spinal Muscular Atrophy Clinical and Preclinical Analyses

**DOI:** 10.1371/journal.pone.0024269

**Published:** 2011-08-31

**Authors:** Dione T. Kobayashi, Rory J. Olson, Laurel Sly, Chad J. Swanson, Brett Chung, Nikolai Naryshkin, Jana Narasimhan, Anuradha Bhattacharyya, Michael Mullenix, Karen S. Chen

**Affiliations:** 1 Spinal Muscular Atrophy Foundation, New York, New York, United States of America; 2 Enzo Life Sciences Inc., Ann Arbor, Michigan, United States of America; 3 PharmOptima LLC, Portage, Michigan, United States of America; 4 PTC Therapeutics Inc., South Plainfield, New Jersey, United States of America; Emory University, United States of America

## Abstract

**Objectives:**

Genetic defects leading to the reduction of the survival motor neuron protein (SMN) are a causal factor for Spinal Muscular Atrophy (SMA). While there are a number of therapies under evaluation as potential treatments for SMA, there is a critical lack of a biomarker method for assessing efficacy of therapeutic interventions, particularly those targeting upregulation of SMN protein levels. Towards this end we have engaged in developing an immunoassay capable of accurately measuring SMN protein levels in blood, specifically in peripheral blood mononuclear cells (PBMCs), as a tool for validating SMN protein as a biomarker in SMA.

**Methods:**

A sandwich enzyme-linked immunosorbent assay (ELISA) was developed and validated for measuring SMN protein in human PBMCs and other cell lysates. Protocols for detection and extraction of SMN from transgenic SMA mouse tissues were also developed.

**Results:**

The assay sensitivity for human SMN is 50 pg/mL. Initial analysis reveals that PBMCs yield enough SMN to analyze from blood volumes of less than 1 mL, and SMA Type I patients' PBMCs show ∼90% reduction of SMN protein compared to normal adults. The ELISA can reliably quantify SMN protein in human and mouse PBMCs and muscle, as well as brain, and spinal cord from a mouse model of severe SMA.

**Conclusions:**

This SMN ELISA assay enables the reliable, quantitative and rapid measurement of SMN in healthy human and SMA patient PBMCs, muscle and fibroblasts. SMN was also detected in several tissues in a mouse model of SMA, as well as in wildtype mouse tissues. This SMN ELISA has general translational applicability to both preclinical and clinical research efforts.

## Introduction

Spinal Muscular Atrophy (SMA) is a progressive neuromuscular disease typified by severe proximal weakness and degeneration of alpha motor neurons in the anterior horn of the spinal cord [1], [2]. SMA is a relatively common monogenetic disorder among rare diseases with a carrier rate of 1 in 35 to 1 in 50 and incidence of 1 in 6000 to 1 in 10000 live births, with a majority of cases presenting in childhood [2]–[4]. SMA is the foremost cause of infantile death among genetic disorders, though the natural history of the disease is evolving due to changes in patient management. Clinically SMA presents as a spectrum of phenotypes, with the most severe cases manifesting symptoms by six months of age with the child never gaining the ability to sit independently and often resulting in death (Type I). SMA patients with milder disease have later onset that presents in between six months of age to the third or fourth decade of life and achieve higher levels of motor function, gaining the ability to sit (Type II) or stand (Type III) though over time patients may progressively lose function and may display characteristics of more severe SMA. The variability in clinical manifestation of SMA is driven by the unique genetics that give rise to the disease. SMA is caused by deletion or mutational inactivation of the Survival of Motor Neuron 1 (SMN1) gene. Humans also carry a second nearly identical copy of the SMN gene called SMN2 [5]. Both the SMN1 and SMN2 genes express SMN protein, however, the amount of functional full-length protein produced by SMN2 is much less (by 70–90%) than that produced by SMN1 [5]–[7]. The SMN2 gene features a C-T replacement in exon 7 that preferentially promotes an alternative splicing pattern that excludes exon 7, resulting in production of an unstable, partially functional truncated SMNΔ7 protein [8]. Although SMN2 cannot completely compensate for the loss of the SMN1 gene, patients with milder forms of SMA generally have higher SMN2 copy numbers, and this phenomenon has been recapitulated in several transgenic mouse models of SMA [9], [10]. This inverse relationship between SMA severity and SMN2 expression provides a strong biological rationale for developing SMA therapeutics that upregulate SMN expression.

Currently SMN upregulation is being aggressively pursued as an SMA therapeutic approach by several investigators. These approaches span the gamut from histone deacetylase (HDAC) inhibitors (e.g. valproic acid, phenylbutyrate, LBH589, SAHA and trichostatin A) that act via multiple mechanisms to increase SMN levels [11]–[17], small molecules that promote the inclusion of SMN2 exon 7, read-through of the aberrant stop codon in the SMNΔ7 mRNA or promote SMN transcription (e.g. aminoglycosides, hydroxyurea, salbutamol, SMN2 antisense oligonucleotides, tetracycline derivatives) [18]–[21], gene replacement of SMN1 [22]–[24], proteasome inhibitors (e.g. MG132, bortezomib ) [25] or other mechanisms (e.g. D157495, hydroxyurea, indoprofen analogues) [26]–[31]. While proof of concept for SMN protein increases have been achieved experimentally in fibroblast and other cell lines derived from SMA patients and even in transgenic SMA mouse models for several of these studies, in the majority of cases SMN levels are being quantified using western blot analysis. Western blot analysis is both time consuming and not fully quantitative, making it a less than ideal tool for assessing SMN levels.

As some of these SMN-targeting approaches evolve into preclinical drug candidates and ultimately enter clinical trials a critical need for a SMN protein measurement tool that is rapid, quantitative, permits a greater throughput for sample testing and is manufactured using standardized processes to maximize reproducibility of measurements has emerged. A number of useful SMN immunoassays have been reported and shown to detect differences in SMN levels in blood and/or fibroblast cells from SMA patients, and to detect therapeutic-induced changes in SMN levels in similar tissues [32]–[34]. While they are of great potential value for drug development, the previously reported assays have not been scaled up for production or validated for use in SMA animal models, and represent a gap in the translation of efficacy results in preclinical testing to measurements in human tissues.

Here we describe the development and application of an SMN enzyme-linked immunosorbent assay (ELISA) that uses a recombinant human SMN protein standard and a capture antibody commonly used in SMA research. The sensitivity of the assay is 50 pg/mL for human SMN. It has been validated for detection of SMN in human peripheral blood mononuclear cells (PBMCs) and has been used to measure SMN protein in brain, spinal cord, liver heart, skin and muscle tissues in a widely used transgenic mouse model of severe SMA. Furthermore, SMN protein was measured in mouse PBMCs, providing a template for analysis and comparison of SMN levels that could bridge drug-induced SMN increases in peripherally available tissue to inaccessible human tissues in clinical studies. Lastly we demonstrate the ability of the assay to measure SMN reliably in human tissue homogenates, as well as in treated cultures from SMA patients, broadening the potential utility of the assay for use in clinical trials and examinations of the natural history of SMA. While the precise relationship between SMA severity and SMN protein levels remains to be defined in these tissues, the SMN ELISA offers the opportunity to address this and other questions of fundamental biology and drug-specific pharmacodynamics of SMN expression.

## Results

### Human SMN protein standard

Recombinant human SMN was generated from full-length cDNA expressed in E. coli containing the pET28a/hSMN-His/TEV vector and purified for use as a standard in the ELISA. After inducing hSMN expression with ITPG, cells were harvested and lysed, with further treatment to extract hSMN from inclusion bodies within cell pellets. The resulting suspension was purified and concentrated over a HiTrap chelating column charged with NiCl_2_. The recombinant protein was greater than 95% pure as determined by SDS-PAGE and densitometry measurements (data not shown), and was used as a reagent in subsequent ELISA experiments.

### Optimization of assay conditions and reagents

Several conditions for optimized SMN signal detection were explored. These parameters included plate coating buffer pH, antibody coating concentrations, and incubation times. The basic reagents used in early optimization experiments were the Sigma 2B1 antibody for the capture antibody, the SC-15320 antibody for the detection antibody and a recombinant SMN protein as the ELISA standard. Conditions that produced the greatest SMN signals in each experiment were selected and incorporated into the protocol for each optimization analysis that followed.

The coating titration analysis indicated maximal efficiency using 3.5 µg/mL of 2B1 with the using the 10 mM PO_4_ 15 mM NaCl pH 7.2 coating solution (Figure S1, S2). The detection antibody was titred to determine the best signal-to-noise ratio for the standard. A detection antibody concentration of 1.5 µg/mL was selected for further testing (Figure S3). Subsequently, both antigen and detection antibodies were tested for 1 and 2 hour incubations, with 30 minute incubations in peroxidase-conjugated goat anti-rabbit secondary antibody and TMB substrate. As no benefits were seen from longer incubations, antigens and detection antibodies were used with 1 hour incubations in all subsequent tests (data not shown). Several reagents were assessed for the ability to interfere with the assay and SDS and sodium deoxycholate were found to strongly interfere with the SMN measurements in the assay. The interference results are summarized in Table S1.

Detection antibodies from Santa Cruz Biotechnology, Novus Biologicals, ProteinTech, and Aviva Systems Biology were tested. Other commonly used SMN antibodies were not tested due to similarities in their SMN N-terminal epitope to 2B1 antibody or to the 2B1 mouse antibody isotype (IgG1). The reactivity to the recombinant human SMN protein was similar between the Aviva and the SC-15320 antibodies while the Novus antibody did not react to the antigen; however, the ProteinTech antibody had 4-fold greater reactance to the protein standard (Table S2). As all of the antibodies reacted to HeLa lysates with the same affinity, the ProteinTech antibody that was most sensitive to the SMN protein standard was chosen for further experiments.

Three extraction buffers were tested with frozen PBMCs that were pelleted and resuspended at 1 mL of buffer per 10^8^ cells. PBMC lysates were tested against the human SMN standard protein at 0.063 to 4 ng/mL for the ProteinTech antibody 11708-1-AP and at 0.25–16 ng/mL for the SC-15320 antibody. The greatest SMN signal with the lowest variability and broadest dynamic range was seen with the combination of ER4 and the ProteinTech antibody (Table S3).

### SMN ELISA reagents and PBMC lysis

The SMN ELISA utilizes a mouse monoclonal antibody raised from immunization with full length SMN protein (2B1), an affinity-purified rabbit polyclonal antibody raised against a GST-tagged SMNΔ7 protein (11708-AP-1), and a recombinant human SMN protein standard. The 2B1 antibody is immobilized on the plate surface and binds with high affinity to the N-terminus within the first 27 amino acids of the SMN protein [35]. The 11708-1-AP antibody binds to the captured SMN and is subsequently detected using a horseradish peroxidase-conjugated goat anti-rabbit polyclonal antibody. Prior to the assay, PBMCs are lysed in a detergent containing buffer and diluted into an assay diluent. A summary of ELISA characteristics is given in Table 1.

**Table 1:** Summary of SMN ELISA Characteristics.

**hSMN Standard Curve**	Dynamic range	3200-50 pg/mL
	Reproducibility	<11% CV
	Sensitivity limit	50 pg/mL
**Freeze Thaw**	Thaw 1 recovery	102%
	Thaw 2 recovery	112%
	Thaw 3 recovery	79%
**PBMC Dilution Linearity**	1∶4	88–106% (98%)
	1∶8	93–109% (102%)
	1∶16	95–112% (105%)
	1∶32	100%
**PBMC SMN Spike Recovery**	267 pg/mL spike	88–116% (100%)
**(1∶4 PBMC dilution)**	667 pg/mL spike	95–137% (105%)
	1667 pg/mL spike	79–131% (99%)
**Minimum Sample Dilutions**	Human PBMCs	1∶4
	Human Muscle	1∶5
	Mouse, Non-Human Primate PBMCs	1∶4
	Mouse Brain, Fat, Heart, Liver, Muscle, Skin, Spinal Cord	1∶5–1∶20
	Mouse Brain Subregions and Sciatic Nerve	1∶4–1∶20

The hSMN Standard Curve: Dynamic range was generated using N = 6 curves from N = 3 experiments. Reproducibility ranged from 4.3–10.6% CVs. The sensitivity limit was derived from the extrapolated value of SMN signal taken at 2 standard deviations above the background level of the assay. Freeze Thaw: The freeze thaw experiment was conducted using a single normal human PBMC sample with a 1∶4 lysate dilution. No reduction in SMN signal was seen with up to two freeze thaw cycles. Recovery was calculated as the mean percentage of SMN signal from a series of up to three sequential freeze thaw cycles compared to the signal from the sample analyzed at the time of cell lysis. PBMC Dilutional Linearity: PBMCs were lysed and diluted from 1∶4 to 1∶32 to assess linearity of sample signal upon dilution. Dilution values were ±5–12% for all analyses with means ranging from 98 to 105%. PBMC SMN Spike Recovery: Using a 1∶4 lysis dilution PBMC samples were spiked with 0, 267, 667 or 1667 pg/mL of the recombinant human SMN used as a standard in the ELISA. The SMN spike signal recovery was calculated based on the expected value from adding the additional SMN to the value produced by the non-spiked samples. Values ranged by +5–37% over the non-spiked signal with mean values ranging from 99–105%, suggesting that accurate analysis of SMN in samples with increased SMN levels is possible, and may benefit from greater sample dilutions. The SMN spike was added into the PBMC mixture at the time of lysis. Minimum Sample Dilutions: Given the differences in sample masses and protein concentrations, different tissue dilutions of µL lysis buffer/mg tissue were used for the SMN protein analysis ranging from 1∶2 to 1∶20.

### SMN detection, assay linearity and recovery in donor PBMCs

**Figure 1:**
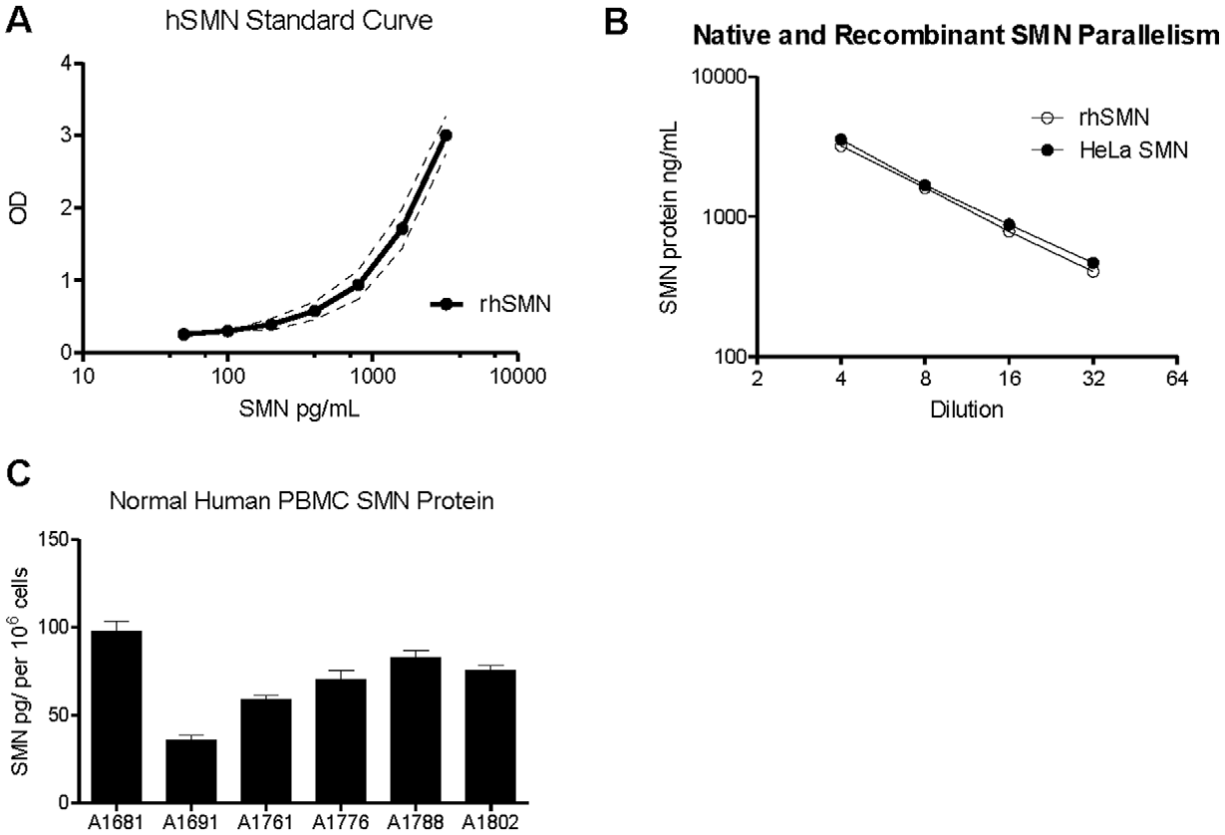
ELISA Performance, parallelism and detection of SMN in human PBMCs. **A**: The recombinant hSMN standard curve data developed from N = 6 curves were highly reproducible with standard deviations of about ±0.23 OD unit variations. The dotted lines surrounding the dose-response curve represent 2 standard deviations. **B**: Comparison of SMN signal detection between recombinant human SMN (dilutions from 1∶4 to 1∶256) and SMN extracted from HeLa cell lysates (1∶4–1∶32) revealed a high degree of parallelism between reagents, allowing for accurate evaluation of native SMN protein using the recombinant SMN ELISA standard. **C**: The SMN ELISA detected protein in adult donor PBMCs; values ranged from 86 to 229 pg SMN per well at a dilution of 1∶4 and an average of 70.2 pg SMN protein per 10^6^ cells. 10^7^ PBMCs diluted 1∶4 through 1∶32 produced linear SMN protein levels. Even with only N = 6 normal samples, SMN levels in PBMCs varied by nearly 3-fold. Error bars represent standard deviations.

The SMN ELISA was run under optimized conditions with the SMN protein standard to determine interassay variability and assay sensitivity. SMN levels were calculated using 3 separate experiments (N = 6) with the recombinant hSMN standard titrated from 0 to 3200 pg/mL (Figure 1A, Table 1). The results from the assays indicate that the lower detection limit or sensitivity of the assay is 50 pg/mL, as this is the concentration two standard deviations above background (Table 1). In addition the interassay variability was low, with coefficients of variance (CVs) between 4.3 and 10.6% (Table 1). HeLa lysates were serially diluted 1∶4 to 1∶512 in assay buffer from a starting undiluted concentration of 1×10^6^ HeLa cells/mL and compared to the same dilutions using the hSMN standard. SMN protein levels were detected in all samples and dilutions with the signal being linear in the 1∶4 to 1∶32 range. Parallelism between HeLa lysate dilutions from 1∶4 to 1∶32 and dilutions of the recombinant hSMN standard was observed, indicating that the antibody binding characteristics of lysates and the standard are similar and the assay SMN protein concentration calculation in the assay is accurate (Figure 1B). Six separate lots of healthy donor PBMCs were acquired (AllCells lots A1681, 1691, A1761, A1776, A1788, A1802) and counted, and cell titrations from these lots were analyzed for SMN protein using the ELISA (Figure 1C). SMN protein levels varied by nearly 3-fold between individuals and the average value for the samples were 70.2 pg/10^6^ cells.

### Validity and comparability of SMN signal detection

**Figure 2:**
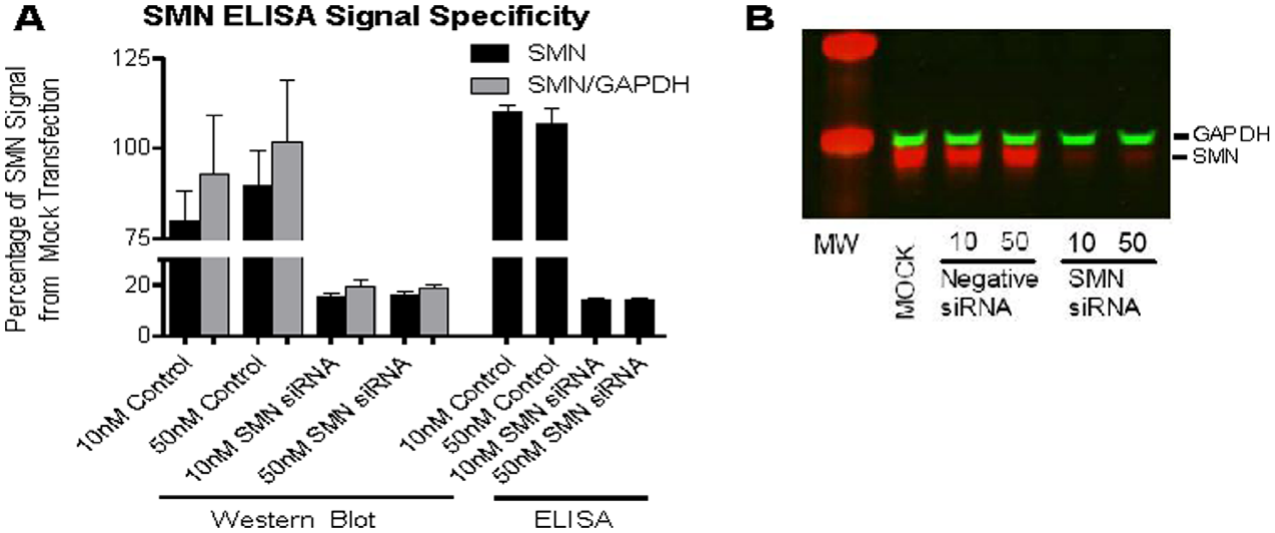
Specificity of SMN ELISA signal detection. To confirm that ELISA signals are specific to SMN protein, SMA Type I fibroblasts were treated with siRNA to knock down the expression of SMN protein. Treated cells were lysed and read in the ELISA and an SMN Western blot. **A**: GM03813 SMN protein levels are reduced by 80–86% compared to mock transfected cells in both Western blot and ELISA analysis. **B**: The image of the SMN Western blot visually corroborates the reduction in SMN levels induced by siRNA knockdown.

To confirm the specificity of SMN signal detection by the ELISA, SMN protein was quantified in GM03813 cells in both the ELISA and western blots after siRNA knockdown. SMN levels were comparable between Western blot and SMN ELISA (Figure 2), confirming the specificity of the SMN signal detected by ELISA in these cells. When quantified as percentage of protein versus mock transfection or as a ratio of SMN to GAPDH signal in Western blot, the SMN levels were reduced by 80–85%; ELISA SMN levels were reduced by 86% of the mock transfection levels (Figure 2A). While no obvious dose-effect was seen from 10 and 50 nM siRNA knockdown, error bars were observed to be smaller with the ELISA analysis.

**Figure 3:**
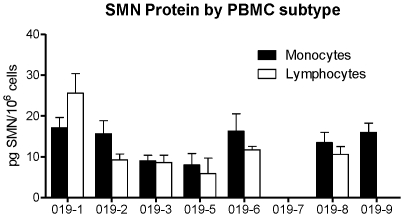
Quantitation of SMN by PBMC subtype. There was no significant difference in SMN protein levels between human lymphocytes and monocytes fractions separated from PBMCs. SMN protein was detectable in the majority of samples for both cell types, ranging from 6.8–25.8 pg/10^6^ cells for lymphocytes and 8–17.5 pg/10^6^ cells for monocytes. Error bars represent standard deviations.

Lysates from PBMC donor lots were used to measure recovery of spiked recombinant SMN, to evaluate native SMN resistance to freeze and thaw conditions, and to determine levels of native SMN in monocyte and lymphocyte subpopulations. In the spike and recovery experiment, recombinant hSMN antigen at concentrations of 267, 667 and 1667 pg/mL were added to PBMC lysate samples diluted 1∶2 and 1∶4 in assay buffer and run in the assay. The recoveries were not reproducible in 1∶2 diluted cell lysates (data not shown). The mean recovery was 101% using a 1∶4 diluted cell lysate (Table 1). PBMC lysates were also tested for stability after multiple freeze-thaw cycles. PBMC lysate from donor A1761 was assessed for SMN protein stability after 0, 1, 2, and 3 freeze thaws (Table 1). After 3 freeze thaw cycles a 21% decrease in protein detection was observed, suggesting that PBMC lysates can withstand up to 2 freeze-thaws without an impact on SMN detection. To determine if PBMC subpopulations express different levels of SMN, PBMCs were separated into lymphocyte and monocyte populations using Miltenyi magnetic beads prior to analysis. The average SMN levels for lymphocytes and monocytes were 9.1 and 12.2 pg SMN per 10^6^ cells respectively (Figure 3). The data were not statistically different suggesting no discernable difference in SMN levels in lymphocytes and monocytes.

Given the results from the cell titration and recombinant hSMN spiking experiments, 1∶4 was chosen as the minimum PBMC lysate dilution out of extraction buffer for subsequent experiments.

### SMN protein in Type I SMA patient PBMCs

The results from the analysis of 6 lots of healthy adult donor PBMCs and subsequent assay validation studies indicated that SMN ELISA could detect SMN protein in lysates from as few as 312,000 cells in healthy donor cells, and that the assay could detect down to 3.6 pg of hSMN per well. Based upon the assay limits of detection, the ELISA would likely reliably detect SMN levels that were as low as ∼10% of the amount found in the average healthy PBMC donor. Therefore the assay was predicted to be capable of measuring SMN in at least some samples from the SMA Type I patients who are expected to have the lowest levels of SMN.

**Figure 4:**
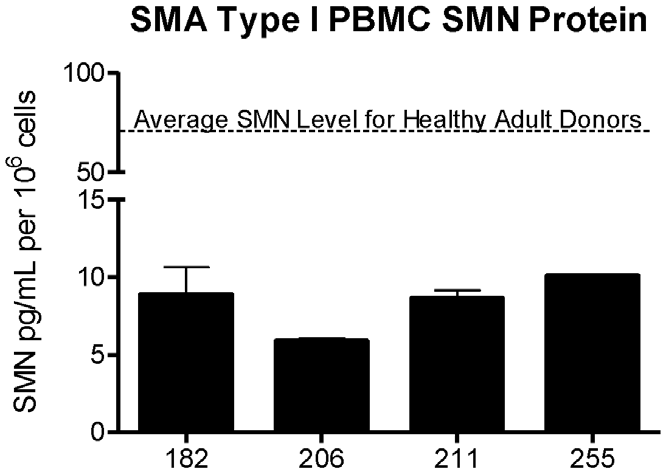
Detection of SMN Protein in SMNA Type 1 PBMCs. SMA Type I patient samples (N = 4) were tested in the SMN ELISA at dilutions of 1∶4 with 1.25×10^6^ and 2.5×10^6^ PBMCs. SMN protein signal was detected in all samples, with an average of 8.32 pg SMN protein per 10^6^ cells. Based on the average of 70.2 pg SMN protein per 10^6^ cells calculated for adult normal donor PBMCs, the amount of SMN protein in PBMCs of Type I SMA patients in this patient cohort is 88% less than normal. Error bars represent standard deviations.

To confirm the ability of the assay to detect the low levels of SMN expected in SMA, 0.5 mL aliquots of PBMC samples in freezing medium were obtained from University of Utah (kindly provided by Dr. Kathryn Swoboda). The samples represented a spectrum of severe disease in Type I SMA and were collected from children who ranged in age from one day through nine months of age (Sample #182, 206, 211,255). Cell counts from the samples ranged from 4.05×10^6^ to 7.6×10^7^ cells. All samples were lysed with 1 mL of lysis buffer with the exception of sample #211 which had a low count and was lysed in 0.5 mL of lysis buffer. As shown in Figure 4, the average SMN level in the 6 healthy adult donors was 70.2 pg SMN/10^6^ cells. In contrast the average SMN protein level from SMA Type 1 PBMCs was 8.3 pg/ 10^6^ cells (Figure 4), only 12% of levels in healthy adult PBMCs.

### Detection of SMN in human buccal cells, saliva, and urine

Several other human fluids and tissues were acquired and tested to determine if the ELISA could detect SMN signals in other peripherally accessible matrices. Evaluation of plasma, saliva and urine for SMN protein did not produce detectable signals at any dilution, while the PBMCs from the same donors yielded signals in the expected range for that matrix (3.4–10.4 ng/mg).

### Measurement of SMN in human muscle

Four skeletal muscle samples from 3 individuals were homogenized in ER4. One sample was from a 37 year old male (#6181A1, unknown muscle type), 2 samples from a 69 year old female (#9834B1 tibialis anterior and #9846A1, unknown muscle type) and 1 sample from a 97 year old female (#7103A1, diaphragm) were prepared at 1∶5 dilutions. In samples #6181A1 and #7103A1, fatty material was visible in the muscle before and after homogenization. Protein concentrations ranged from 1.36 to 6.92 mg/mL and the two samples with greater fat content had the two lowest yields of total protein (Table 2). SMN protein was detectable in all homogenates, with concentration ranging from 454 to 2230 pg/mL (Table 2). The concentration of desmin, a muscle-specific protein, was also quantified using an ELISA for human desmin and the concentration ranged from 1.39 to 18.5 ng/mL. SMN levels were normalized to either total soluble protein or desmin and both normalized data agreed with each other, suggesting that neither measurement presents an obvious advantage for normalizing SMN levels.

**Table 2:** SMN protein in human muscle.

Sample	High fat content?	Total protein (mg/mL)	SMN (pg/mL)	Desmin (ng/mL)	SMN pg/mg total protein	SMN pg/ng Desmin
6181A1	Yes	4.53	454	18.5	501	24,500
7103A1	Yes	1.36	810	1.39	2980	583,000
9834B1	No	6.92	2230	8.48	1620	263,000
9846A1	No	5.94	693	9.24	582	75,000

### Detection of SMN changes in an SMA patient fibroblast line

**Figure 5:**
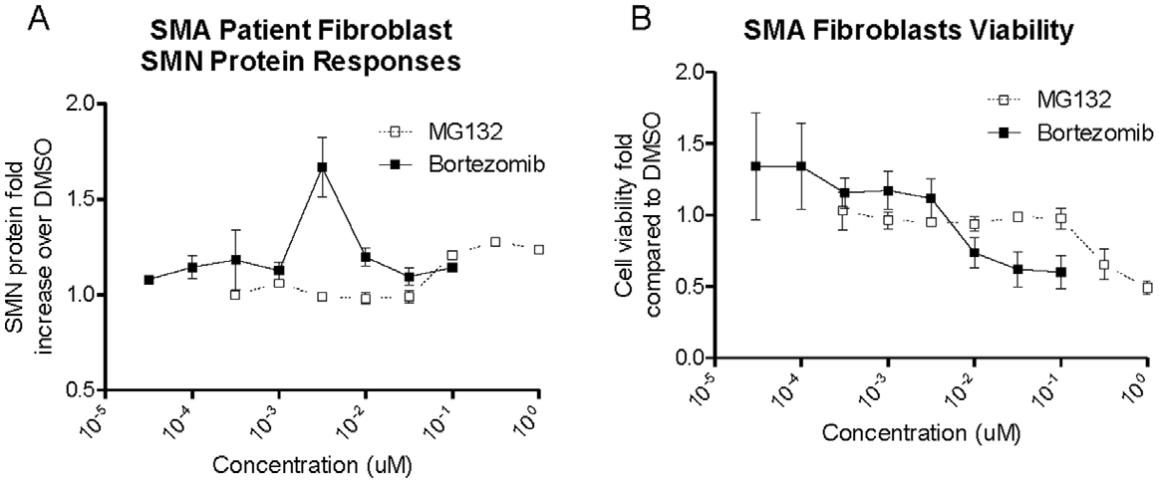
Detection of SMN protein dose-response in SMA fibroblasts. SMA Type I fibroblasts (line GM03813) were treated for 24 h with the proteasome inhibitors MG132 or bortezomib (N = 2 each). **A**: A peak SMN protein upregulation of 27% was observed with 0.3 µM of MG132 (EC_50_ = 0.68 uM) and a 67% increase was seen with 3 nM bortezomib (EC_50_ = 10 nM). **B**: Cell viability as measured by ATP levels (CellTiter-Glo, Promega) decreased to 50–75% at doses near and above doses that increased SMN protein levels. Error bars represent standard deviations.

SMN upregulation was detected in Type I SMA fibroblasts (GM03813) treated with proteasome inhibitors MG132 and bortezomib (Figure 5). Serially diluted compounds were added into the cell culture medium and incubated for 24 h in the presence of 0.001 to 1 µM MG132 or 0.1 to 100 nM bortezomib in 0.5% DMSO. SMN protein was measured using the SMN ELISA. A 1.27 fold increase in SMN protein over signal from DMSO-treated cells was observed in fibroblasts treated with 0.3 µM MG132 whereas 3 nM of bortezomib produced a 1.67 fold SMN protein increase (Figure 5A). Both MG132 and bortezomib were found to be cytotoxic to GM03813 fibroblasts, a (Figure 5B).

### Reactivity of human SMN is greater than that of mouse Smn in the ELISA

**Figure 6:**
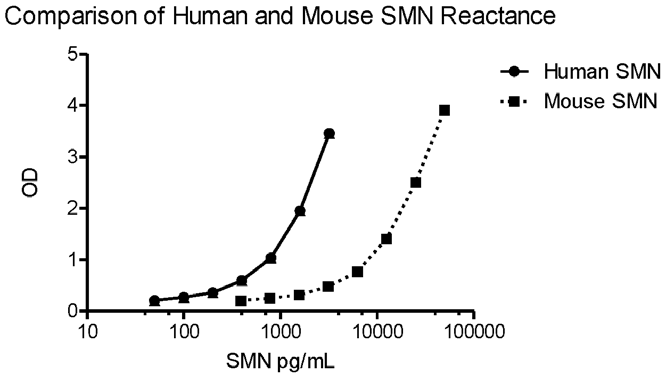
SMN species parallelism. Full-length recombinant mouse and human SMN proteins were tested in the ELISA at a range of concentrations (390–50000 pg/mL and 50–3200 pg/mL respectively). The human SMN was detected with 10-fold greater signal at all points than the mouse SMN. The relationship between the mouse to human dose-response curve was linear and values were comparable across equivalent points in the dilution curves (p = 0.86). Error bars represent standard deviations.

Quantification of recombinant mouse and human SMN proteins using the ELISA in the 390 to 50000 pg/mL and 50 to 3200 pg/mL range respectively showed a marked difference in reactivity to the SMN protein between the two species. Recombinant mouse Smn was only detected at ∼10% the levels of recombinant human SMN on a concentration basis. However, despite the lower reactivity, the mouse SMN signal is still linear in the dynamic range of the assay (Figure 6).

### SMN signal across species

**Figure 7:**
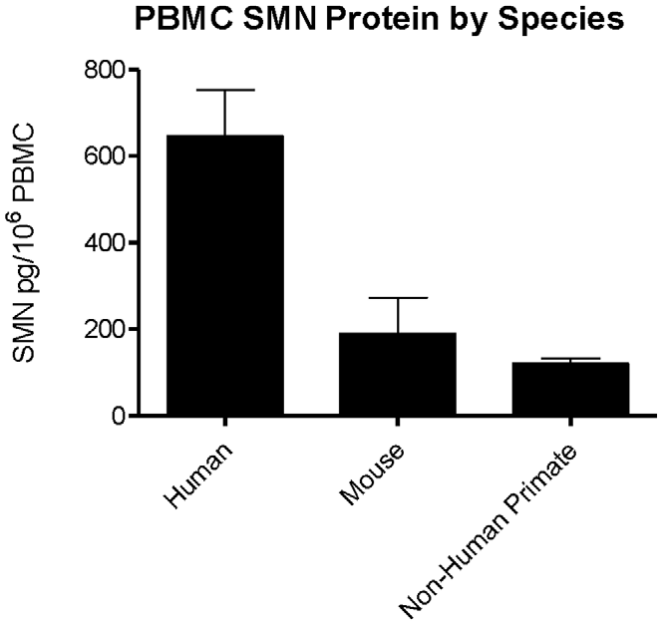
ELISA SMN signal detection across species. SMN protein was detected in human, primate and mouse in the ELISA. All samples had viabilities of 80–95%. Error bars represent standard deviations.

SMN protein signal from human, wild-type mouse, and non-human primate (NHP) PBMCs (N = 1–2) was assessed in the ELISA at dilutions of 1∶4 to 1∶32. Due to the high numbers of PBMCs in the human and NHP samples, cells were diluted at a ratio of 1 mL/10^7^ PBMCs in the ER4 buffer. Cell viability upon thawing was between 70–95%. Human, NHP, and mouse signals were detected across all dilutions and yielded 646, 121 and 190 pg/10^6^ PBMCs respectively (Figure 7). The SMN levels seen in this experiment were about 10-fold higher than the ones seen from PBMCs lysed at 1 mL/10^8^ cells (Figure 1C, Figure 6).

### SMN protein quantification and timecourse in a severe model of SMA

**Figure 8:**
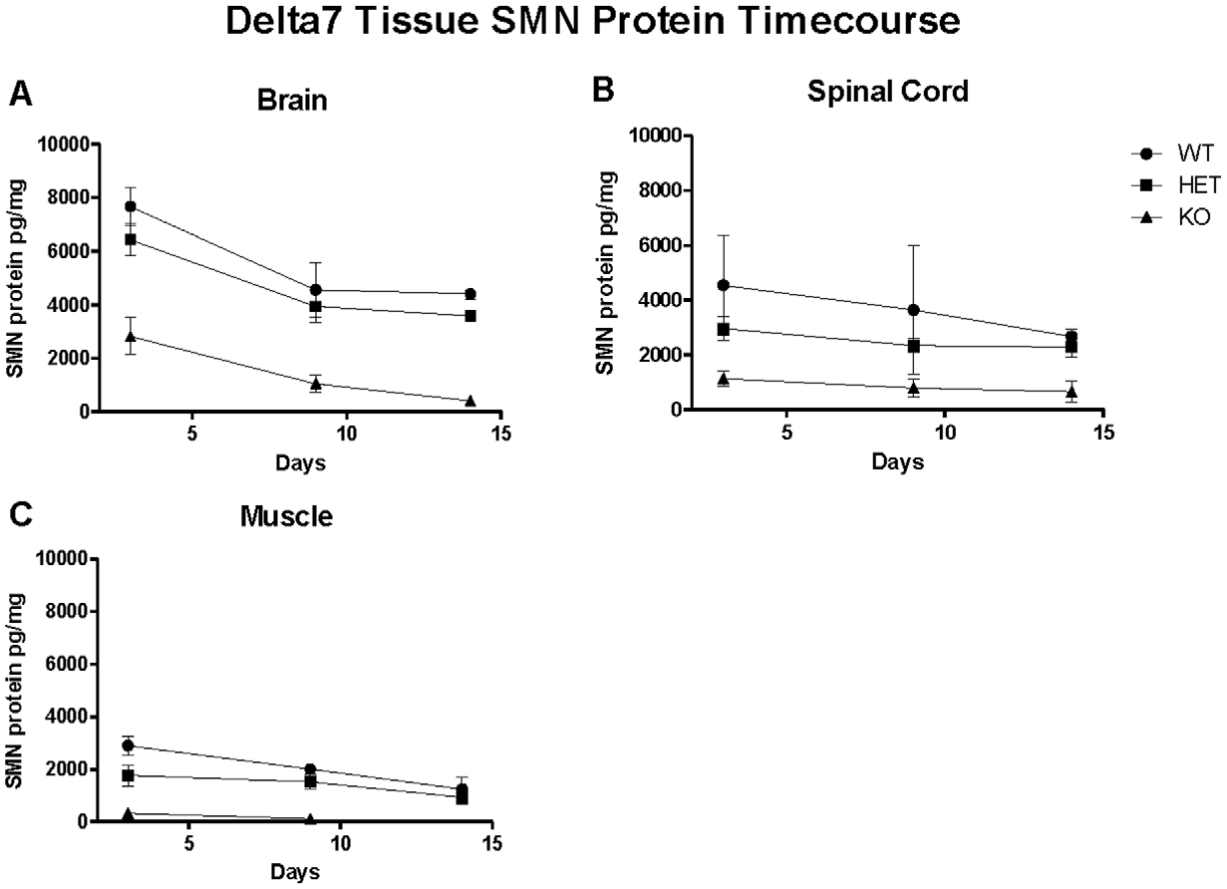
SMN protein in WT, HET and KO Delta7 mice was detected by the ELISA. While tissue levels in brain homogenates were higher than both spinal cord and muscle across all genotypes, the ratio of brain to spinal cord, and brain to muscle in the homozygous KO mice was 1.4-fold and 12.2-fold, respectively. The decline of SMN levels over time between muscle and spinal cord tissues was relatively similar across genotypes; however, the reduction seen for brain SMN was more precipitous dropping ∼40% between P3 and P9. Variability was greatest in the spinal cord tissues, and SMN levels in KO muscle tissue approached the limit of detection by P9. Statistical analyses are presented in Table 2. Error bars represent standard deviations.

The SMN ELISA enabled quantitative measurement of SMN levels across ages in a severe model of SMA. The Δ7 model is a commonly-used model of severe SMA disease with a deletion of the mouse Smn gene, a complete human SMN2 transgene that predominantly generates an SMN2Δ7 transcript lacking exon 7, and multiple copies of the SMN2Δ7 cDNA, which results in a short-lived protein [36]. Brain, spinal cord and muscle were harvested from postnatal day P3, 9 and P14 from Δ7 wild-type (WT), heterozygous (HET) and knockout (KO) mice, (all carrying the human SMN2 and SMN2 Δ7 cDNA genes and, respectively, 2, 1, 0 copies of the mouse Smn gene. Standard deviations were generally low, with the exception of muscle tissue in the KO animals, as the SMN protein levels in these tissues was closest to the limit of detection for the ELISA at P3 and P9 and was not detectable at P14 (Figure 8). Generally SMN levels in KO mouse tissues were less than 10% the levels of SMN protein in WT mouse tissues (Figure 8, Table 3). SMN protein levels decreased in the following order brain>spinal cord>muscle (gastrocnemius) for each genotype at any given age (Figure 8, Table 3). The fold difference in SMN levels between brain and spinal cord was similar in both WT and HET mice (2.3 and 3.8-fold). The SMN expression difference between the brain and muscle was greatest in KO mice (8.5-fold less) than WT and HET (2.3 and 3.8-fold less, respectively) (Figure 8A, 8C). Additionally, the SMN protein levels in KO mice ranged from ∼3 to 10-fold less than those in WT animals at any given age or in any given tissue. Between P3 and P14, the levels of SMN were decreased in all genotypes, suggesting a natural developmental expression change. However, the slopes of the decline in SMN expression over time across genotypes and tissues were not the same, with brain declining the most rapidly of the three tissues. SMN expression levels in the brain decreased by 85% in KO mice from P3 to P14. Spinal cord SMN signal diminished by 43% in the same timeframe (Figure 8B, Table 3). Furthermore, in muscle tissue the SMN protein decline was linear between P3 to P14, while the decline of SMN protein in brain was more precipitous between P3 to P9 than P9 to P14. The variable pattern of decline across tissues and genotypes suggests differential regulation of SMN in the Δ7 model or alternatively different pathological disease processes that may affect SMN expression.

**Table 3:** SMN protein timecourse across Delta7 mouse genotypes with statistical significance.

Genotype	Tissue	Age	SMN pg/mg total protein	SD
**WT**	**Brain**	P3	7660	716
		P9	4550^***^	143
		P14	4400	194
	**Muscle**	P3	2900^+++^	379
		P9	2010* ^+++^	164
		P14	1250^**+++^	441
	**Spinal Cord**	P3	4540^+++^	1820
		P9	3640	1360
		P14	2670^***+++^	247
**HET**	**Brain**	P3	6440	605
		P9	3940^***^	588
		P14	3580	188
	**Muscle**	P3	1770^+++^	403
		P9	1530+	303
		P14	934 ^**+++^	250
	**Spinal Cord**	P3	2960^+++^	444
		P9	2340* ^+++^	259
		P14	2280^+++^	375
**KO**	**Brain**	P3	2820	694
		P9	972^***^	329
		P14	402	151
	**Muscle**	P3	333^++^	83
		P9	122* +	30
		P14	<LOD	N/A
	**Spinal Cord**	P3	1130^++^	259
		P9	789	332
		P14	644	378

SMN protein values generally decline from P3 to P9 and P14 across all genotypes, though the rate of decline is distinct between genotypes. In general SMN protein levels in brain were higher than levels in spinal cord and muscle across all ages and genotypes. An ANOVA with Tukey's post-test performed for all analyses with the exception of the WT and HET comparisons at P14 that were done with an unpaired t-test as at P14 KO muscle signal was below the assay LOD.

*Denotes SMN protein values are significantly less statistically than the value of the same tissue at the previous age.

+ Denotes SMN protein values are significantly less statistically than the value of the brain tissue of the same genotype and age. P-values are indicated by asterisks or plus signs in the following manner: p<0.001 by ***, p<0.01 by** and p<0.05 by *. LOD = limit of detection.

### Expression pattern of Smn protein in PBMCs, CNS and other WT mouse tissues

To establish a template for analysis in target and easily accessible tissues, Smn was measured in several tissues in wild-type FVBn mice (N = 7 at 14 weeks of age). Analyzed tissues included brain, muscle, spinal cord as well as liver, heart, abdominal adipose tissue and ear pinnae and PBMCs. Whole blood was extracted from the same mice in a terminal collection. A Ficoll gradient was used to isolate PBMCs. The volume of whole blood collected from the mice ranged from 0.350 to 0.700 mL, and total viable cell counts ranged from 3 to 14.8×10^6^ cells/mL of lysate (0.8 to 2.5×10^6^ cells/mL whole blood). Viability of cells ranged from 95 to 99%. Concentrations of Smn in tissue samples were determined based on a standard curve derived from recombinant human SMN.

**Figure 9:**
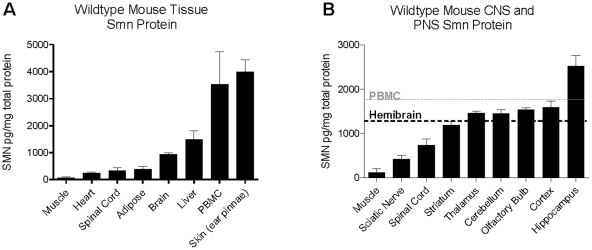
Smn protein levels by tissue in WT mice. Smn protein levels are distinct across the an array of tissues and regions of the brain. **A**: Smn levels varied by as much as 10-fold across tissue types in adult FVB mice (14 weeks old). SMN levels by tissue were distributed on a basis loosely ordered by having lesser to greater dividing cell populations. Smn levels for tissues are represented as Smn pg/mg total protein. PBMCs are represented as Smn pg/mg total protein in the lysis buffer extract. On a per cell basis the average Smn level was 67.2 pg/10^6^ PBMCs. **B**: Smn protein in the brain, spinal cord, sciatic nerve and quadriceps muscle of wildtype mice shows marked variations, with spinal cord having 6-fold greater levels than muscle and nerve. In the brain, Smn protein signal in the thalamus was nearly 2.5-fold less than hippocampal levels. Error bars represent standard deviations.

Mouse Smn was detectable in all tissues and PBMC samples (Figure 9A). The average Smn levels in mouse PBMCs were calculated using either per mg of total soluble protein (3.5 ng/mg total protein) or per 10^6^ cells (67.2 pg/10^6^ cells). Despite differential reactivity to the recombinant human and mouse SMN protein (Figure 6), the amount of Smn detected in wild-type mouse PBMCs was similar to that found in healthy humans at 70.2 and 67.2 pg/10^6^ cells (Figure 1C, 9A). Surprisingly, the levels of mouse Smn detected across the panel of tissues ranged 60-fold from the lowest expressing tissue (muscle) to the highest (ear pinnae skin) (Figure 9A). Generally there appeared to be three strata of Smn levels by tissue, with the lowest levels in the muscle (66 pg/mg), heart (251 pg/mg), spinal cord (329 pg/mg), and adipose tissues (389 pg/mg). SMN levels in liver and brain were intermediate at 933 and 1110 pg/mg respectively, and the highest levels were observed in PBMCs and the ear skin (3980 pg/mg).

Smn signal was detected in mouse hemibrain, spinal cord, quadriceps and sciatic nerve processed via Precellys homogenization (Figure 9B). Muscle and nerve had the lowest levels of this tissue set, at 119 pg/mg and 425 pg/mg respectively. Smn varied by over 2-fold across regions of the brain, from 1192 pg/mg in the striatum to 1536 to 1587 pg/g in the olfactory bulb and cortex, to their highest levels in the hippocampus measured at 2520 pg/mg on average (Figure 9B).

### Comparability of SMN protein measurements in mouse tissue

To confirm the relationship between SMN protein as detected by the ELISA and another quantitation method, HET and KO Delta7 mice were tested in the ELISA and Western blot. Results between the two methods were similar as the ratio of SMN signal and total protein between HETs and KOs was 2.2-fold in the ELISA and 2.3-fold in the Western blot (Supplementary Figure S4).

## Discussion

This report describes the development of a sensitive and reliable SMN ELISA tool that has utility in preclinical studies and imminent new clinical trials in SMA. The 50 pg/ml detection limit of the assay permits analysis of SMN levels in human and mouse PBMCs and muscle, human fibroblasts and several other tissues in WT and SMA model mice. Other efforts have also produced useful SMN ELISAs [32], [33], [37]. The assay described here is distinct in that it has undergone optimization and quality assurance tests for scale-up and manufacturing and can be used as a kit by clinical laboratories as well as basic researchers. Our findings have implications for the design of both preclinical and clinical studies in SMA with respect to timing, mass and volume of sample collections that may be executed in trials of SMN-upregulating therapeutics. This ELISA also provides a tool for developing preclinical pharmacodynamic information on responses to SMN-upregulating therapeutics that can be translated to human studies by virtue of being done in the same peripherally accessible tissue (PBMCs) between the bench and the clinic.

PBMCs from mice and healthy human donors and SMA patients were assessed for SMN levels successfully using the ELISA. SMN levels were similar in samples from normal human donors and mice at 6.32 ng/mg versus 3.53 ng/mg respectively when prepared in 1 mL/10^6^ cells for lysis (Figures 1C, 9A), but the direct quantitative comparison across species is not possible given the difference in SMN signal in the ELISA between species. PBMCs from very young Type I SMA children had 88% less SMN relative to normal adult donors. These data were derived from a small number of samples but they agree with the data generated using western blots and other SMN immunoassays incorporating different antibodies and measuring SMN in different cell types [9], [27], [32], [37], [38]. In dilution experiments using healthy human PBMCs, SMN was measurable at levels ∼5-fold over the limit of quantitation using 3×10^5^ cells, suggesting that analysis would be possible with far fewer cells or in a smaller blood volume. Given that use of different ratios of lysis buffer to PBMCs yields as much as a 10-fold increase in SMN protein signal (Figure 1C, 7), it is important to explore the lower limits of blood sample volumes as part of the effort to evaluate conditions for PBMC collection and storage processes to maximize detection of SMN for multisite SMA clinical studies.

SMN was readily detectable in human muscle tissue samples and SMA patient fibroblasts (Table 2, Figures 2, 5). Knockdown of SMN in Type I SMA GM03813 fibroblasts with siRNA resulted in 80–86% reductions in protein levels as measured Western blot and ELISA analyses, confirming the validity of the ELISA's signal detection (Figure 5). SMA fibroblasts treated with proteasome inhibitors produced a quantifiable 27–67% increase in SMN protein; the data also underscore the need to normalize SMN levels to cell counts or total protein as the levels were increased at concentrations that also showed cytotoxicity (Figure 5). Analysis of the data suggests that the ELISA can be effectively used to detect a 20% change in fibroblast SMN levels with only a few samples and it may be useful for secondary drug discovery compound screening in a 96-well plate format (Figure 5). The human muscle SMN signal varied widely across samples and further exploration with a more limited set of muscle types and donor ages is warranted. While muscle is important as a possible SMA disease target tissue, the contribution of excess intramuscular fat in SMA muscle samples should be carefully considered for interpreting a muscle-specific SMN signal [39]. While normalization to the muscle marker desmin agreed with SMN normalized to total protein content, normalization against adipose-specific markers may also be useful to explore. Thus, justification for SMA muscle collections in trials for SMN measurement with this ELISA requires further study with more annotated muscle samples.

While these data demonstrate that SMN can be reliably measured in PBMCs, it is important to note the challenges of working with PBMCs and limitations on sample collections inherent to analysis in fragile pediatric SMA patients. PBMCs require complex processing and are sensitive to diverse factors including acute and chronic inflammation. SMA clinical studies utilizing PBMCs will need an optimized protocol for collection, processing and storage and these studies are underway. The allowable volume of whole blood that may be drawn from a child or infant for study is also limited, and because there are likely to be several other blood analyses performed in drug trials, analysis of PBMCs for SMN protein needs to be balanced against other sample uses. Generally, 1×10^6^ PBMCs can be isolated per milliliter of whole blood. The SMN ELISA described here should allow 2 or more analyses per mL of whole blood drawn. Children have been reported to have higher PBMC counts than adults in healthy populations. Higher PBMC counts have not been established in children from SMA population, if confirmed then it becomes possible to test SMN levels with smaller volumes of blood [40].

One important perspective on the analysis of SMN in PBMCs in SMA for the purposes of assessing the efficacy of an SMN-upregulating drug is that the levels of SMN in blood cells may or may not be reflective of SMN levels in disease target tissues in the spinal cord or muscle. In addition to determining the relationship between baseline PBMC and SMA target tissue SMN levels, it is important to determine whether PBMCs will be effective cells for assessing SMN response to therapeutics, and relate the relationship of changes in PBMCs to changes in spinal cord and muscle. As one way of assessing this, we developed a template for analysis in the mouse to determine relationships between SMN levels in peripheral and disease-target tissues in mouse and human which will enable decision making for clinical trial designs.

The ELISA is able to detect the Smn protein in mice, despite differences in reactivity between human and mouse recombinant proteins in the assay, and is able to detect Smn in PBMCs from the *Macaca* genus (Figure 6, 7). It is unclear the extent to which the different reactivity observed between human and mouse is due to differences in the epitopes recognized by the two assay antibodies, or to differences in the expression and purification of the two recombinant proteins. Based on peptide mapping experiments, the epitope for the ELISA's capture and detection antibody are proposed to be between amino acids 14–20 and 197–204 in the human SMN protein, and between amino acids 11–17 and 192–197 in the mouse Smn protein (personal communication, Andreas Weiss, Novartis). The shorter epitope for the mouse protein towards the C-terminus may be playing a role in the differential detection between the two species. As SMN measurements from a macaque species were previously reported by Battaglia et al., these data may motivate researchers interested in measuring Smn from these animals to consider similar polyclonal SMN antibody strategies for measuring Smn in macaques [41]. Furthermore, while commercial sources with validated protocols for collection and processing of porcine PBMCs were not available, it is expected that the ELISA will perform like Western blots with the BD antibody in detecting native Smn in porcine models for large-animal analysis for SMA therapeutics that are in development [42], [43].

The results from the wild-type and SMA model mouse tissues showed striking differences in SMN levels among tissues and generally were comparable to signals detected in Western blots (Supplemental Figure S4). Consistent with other publications, SMN levels were higher in brain than in spinal cord and higher in spinal cord than in muscle. The comparative analysis across tissues provided the novel measurement of up to a 60 fold increase in SMN levels within tissues of the same genotype. SMN levels in mouse PBMCs are intermediate between liver and skin tissues when normalized to total protein levels [24], [36]. It is possible to broadly classify the liver, skin and PBMCs into a group of tissues with high SMN levels that contain a large number of cells capable of rapidly dividing whereas brain, spinal cord, muscle and heart (with the possible exception of the hippocampus and olfactory bulb) are largely post-mitotic organs with smaller populations of dividing cells [44]. Regional differences in brain structures are observable using the ELISA and generally replicate prior reports (Figure 9B) [41], [44]. Analysis of tissues in the complete motor circuit of brain, spinal cord, muscle and nerve produced results similar to that done with a different homogenization method, and it was striking to note that muscle, nerve and spinal cord, the primary tissues associated with SMA, generally had the lowest levels of Smn measured in tissues.

Whether SMA target tissues will respond to drugs designed to increase SMN in the same manner as in other tissues must be determined on a case-by-case basis for each drug, as drug exposure and metabolism across tissues will figure heavily into this answer. The differences in expression levels across tissues motivate speculation that each tissue may tolerate and respond to SMN depletion in different ways. This data also provides a glimpse at the scope of regulatory processing that impacts SMN protein expression. It is of interest to note that the ear skin tissue in WT mouse with highly abundant SMN protein is a tissue that together with the tail and hindpaws becomes progressively necrotic in SMA mice that are rescued from SMN depletion by genetic reintroduction of SMN or therapeutic upregulation of SMN [23], [24], [45], [46]. While the necrosis is very rarely seen in SMA patients [47], [48], and the phenomenon has been hypothesized to be a possible result of impairments to autonomic and/or vascular systems, it is also possible that SMN depletion contributes to this phenotype in tissues with exceedingly high normal expression. These findings do support the emerging thesis in the SMA field that SMN depletion causes an organism-wide disorder that may produce dysfunction in tissues and systems not normally associated with SMA and in turn these tissues may influence the more primarily SMA-like phenotypes of the CNS and musculature [44], [49].

The postnatal decrease in SMN expression seen in WT, HET and KO Δ7 mice is also consistent with other reports and the similarity between SMN ELISA and Western blot results suggest that the ELISA is capable of detecting the highly expressed but short-lived Δ7 protein species [8], [50], [51]. It is clear that the rate of SMN decline is different between brain, spinal cord and muscle. Given the 14-day lifespan of the Δ7 KO mouse, and the severity of the muscle weakness phenotype, it is important to carefully select the time of intervention for SMN-upregulating drug studies and to place the data in the correct context of the developmental SMN protein decline, which differs between tissues.

These SMN ELISA data provide frameworks from which investigators may explore a central question in SMA therapy – how much SMN is needed to rescue function? Given that hindlimb muscle weakness is reported and motor neuron loss in the L3–L6 region is not observed at P4 but is present at P9 in Δ7 mice it is notable that in spinal cord and hindlimb muscle SMN levels decrease by only 20–25% (Figure 8B, 8C, Table 3) [36], [52]. Whether this decrement is evidence that tissues with relatively modest SMN levels are unable to tolerate loss of SMN compared to the brain which loses 50% of its SMN signal in this timeframe, or whether this is cause for optimism in thinking that only modest increases in SMN may be needed to produce benefits in mouse models or human patients is debatable and requires much more investigation.

In summary, SMN protein analysis via a sensitive and reliable ELISA offers a unique opportunity to generate information that is of directly translatable preclinical and clinical value. Fully quantitative analysis of human PBMCs and fibroblasts will allow for determination of SMN responses to drugs both in basic research and drug screening as well as in a clinical trial setting. The SMN protein analysis of human and mouse tissues in the CNS, internal organs, and blood and skin tissues provide a framework for developing drug-response relationships between peripherally accessible tissues and target SMA tissues in animal models that can then be extrapolated to the patient on a drug-by-drug basis. Interestingly, the details of the broadly variable SMN protein expression pattern also suggest a high degree of regulation across tissues, and this may influence both primary and secondary disease mechanisms. While these data are collectively of some interest, their greater value may lie in motivating further exploration of SMN regulation, investigation of SMN dependence across tissues, more quantitative testing of SMN-upregulating drug responses as well as optimization of tissue analysis for SMA clinical trials.

## Materials and Methods

### Ethics Statement

SMA patient samples from the University of Utah were collected in accordance with study protocols approved by the University of Utah Institutional Review Board (IRB) (clinicaltrials.gov registry # NCT00756821), with written and verbal consent obtained. Human samples from Asterand (Detroit, MI) were harvested via autopsies from a network of hospitals and are exempt from IRB approval; next of kin provided written approval for tissue harvest. Samples from Analytical Biological Services (Wilmington, DE) were collected with written informed consent provided by donors or their guardians. Samples from AllCells (Emeryville, CA) were collected from donors who gave written consent in accordance with protocols approved by their governing IRB (Biomed). Primate blood samples from BioQuant (San Diego, CA) were collected in accordance with practices recommended by the Weatherall Report. All animals were maintained and harvested according to protocols approved by each facility's Institutional Animal Care and Use Committee (IACUC). At PharmOptima the animal tissues were harvested under protocol 10-08-02 approved by the Western Michigan University IACUC. Mouse tissues collected at PsychoGenics were harvested under protocol 86-2-0310 approved by the PsychoGenics IACUC.

### PBMC sources and processing

Sample collection was done according to the guidelines of the IRB at the University of Utah. Donor adult PBMCs were purchased from AllCells (Emeryville, CA) and SMA Type I patient PBMCs were generously provided by Dr. Kathy Swoboda of the University of Utah. Samples were collected perinatally to 4 months of age. Whole blood samples at the University of Utah were collected in BD CPT tubes (#362761) and centrifuged at 1600RCF for 30 minutes at 20°C. After removal of plasma, the buffy coat containing PBMCs was placed in a 15 mL tube and filled to 15 mL with PBS and centrifuged at 300 RCF for 15 minutes at 20°C. Supernatant was removed and the pellets were resuspended in 10 mL PBS and centrifuged for 15 minutes at 20°C. After final removal of supernatant, PBMCs were resuspended in freeze media (20% Fetal Bovine Serum, 10% Dimethylsulfoxide in RPMI media), transferred to cryovials and frozen at −140°C until lysis. Sample collection was done according to the guidelines of the Institutional Review Board at the University of Utah.

### PBMC fractionation analysis

The PBMC cell pellet was resuspended in 80 µL assay buffer per 10^7^ total cells, followed by addition of 20 µL of CD14 MicroBeads (Miltenyi #130-050-201, Auburn, CA), per 10^7^ total cells for positive monocyte selection. Cell suspension was mixed and incubated for 15 minutes at 4°C. Cells were washed by adding approximately 2 mL buffer per 10^7^ total cells, followed by centrifugation. Supernatant was decanted and cell pellet was resuspended in 500 µL buffer. The columns were placed in the magnetic field of the OctoMACS™ (Miltenyi Biotec #130-042108) separator. The column was washed with 500 µL buffer. The cell suspension was applied to the column. Unlabeled cells (lymphocyte fraction) that passed through the column were collected. The column was washed three times with 500 µL buffer and collected in the same tube (∼2 mL total effluent per sample). One milliliter of buffer was added to the column, and the magnetically labeled cells were flushed out by plunger.

### Recombinant SMN protein expression and purification

Expression of SMN1 was performed in *E. coli* BL21DE3 cells containing the recombinant plasmid pET28a/hSMN-His/TEV expression vector. The recombinant protein was expressed by adding isopropyl B–D-thiogalactopyranaside (IPTG) at a final concentration of 1 mM. The induced cells were harvested, resuspended in 20 mM sodium phosphate pH 7.4, 500 mM NaCl, 10 mM imidazole, and protease inhibitors and disrupted by sonication. The sonicate was centrifuged and the supernatant discarded. The pellet was resuspended in 6 M urea to denature proteins expressed in inclusion bodies. The suspension was centrifuged and the supernatant collected and loaded onto a HiTrap Chelating HP column charged with NiCl_2_. The column was washed with 20 mM sodium phosphate pH 7.4, 500 mM NaCl, 10 mM imidazole, and 6 M urea and the protein was eluted with 20 mM sodium phosphate pH 7.4, 500 mM NaCl, 250 mM imidazole, and 6 M urea. Fractions containing the protein were pooled and concentrated by running the protein solution back over the column charged with NiCl_2_. Using the same process previously described, the protein was eluted from the column and re-folded by dialysis.

### ELISA reagent and assay conditions optimization

Using the Sigma 2B1 antibody (#S2944, St. Louis, MO) for the capture antibody, the SC-15320 antibody (Santa Cruz Biotechnology, Santa Cruz, CA) for the detection antibody and the recombinant SMN protein standard, several conditions for optimized SMN signal detection were explored. The following coating buffers and pHs were tested: 10 mM PO_4_ 15 mM NaCl pH 7.2, 10 mM PO_4_ pH 8.0, and 200 mM Carbonate-Bicarbonate pH 9.4 using 16 µg/mL of the 2B1 antibody. Subsequently, the 2B1 antibody was plated at 2, 4, 6, and 8 µg/mL using the 10 mM PO_4_ 15 mM NaCl pH 7.2 coating condition. The SC-15320 detection antibody was titrated to determine the concentration that would produce the best signal-to-noise ratio. The human SMN recombinant standard was tested at 0, 0.5 and 16 µg/mL against 62.5–2500 ng/mL of detection antibody, and 1.5 mg/mL was selected for further testing. Antigen and detection antibodies were tested for 1 and 2 hour incubations, with 30 minute incubations in peroxidase-conjugated goat anti-rabbit secondary antibody and TMB substrate. The 1 hr incubation time was used in further analysis.

Several reagents were assessed for the ability to interfere with the SMN protein signal. All interference reagents were tested in assay buffer for signal detection with 16 ng/mL of the recombinant human SMN standard protein and compared to an assay buffer control (100 mM PO_4_, 150 mM NaCl, 1% Bovine Serum Albumin [BSA], 0.1% Tween-20). Ethylenediaminetetraacetic acid (EDTA), ethylene glycol tetraacetic acid (EGTA) and phenylmethanesulfonylfluoride (PMSF) were tested at 1, 2, 4, 8 mM. NP-40 was tested at 2, 4, 8, 16%. PIC8340 was tested at 0.5, 1, 2, 4 µL/mL. Sodium dodecyl sulfate (SDS) was tested at 0.1, 0.2, 0.4, 0.8%, Sodium deoxycholate was tested at 0.06, 0.13, 0.25, 0.50%. Triton X-100 was tested at 0.25, 0.5, 1, and 2%. Zwittergent 3–14 was tested at 0.006, 0.013, 0.025 and 0.05%.

### SMN antibody and extraction buffer screening

The 2B1 antibody from Sigma was chosen as the capture antibody and further optimization was done for SMN protein extraction buffers and SMN detection antibodies. Detection antibodies from Santa Cruz Biotechnology (#SC-15320, Santa Cruz, CA), Novus Biologicals (#NB100-57859, Littleton, CO), ProteinTech Group (#11708-1-AP, Chicago, IL), and Aviva Systems Biology (#ARP40209-P050, San Diego, CA) were tested. Frozen PBMCs were thawed and split equally into 3 conical 15 mL tubes for selection of an optimized SMN extraction buffer. Three extraction buffers were tested:100 mM Tris, pH 7.5, 2.5% NP-40; 100 mM Tris, pH 7.5, 2.5% NP-40, 300 mM NaCl, 0.5% SDS, 25 mM NaF, 3 mM EDTA, 1 mM MgCl2, 20 mM β-Glycerophosphate; and 50 mM Tris, pH 7.5, 300 mM NaCl, 10% (w/v) glycerol, 3 mM EDTA, 1 mM MgCl2, 20 mM β-glycerophosphate, 25 mM NaF, 1% Triton X-100 (ER4). Cells were pelleted and resuspended at 1 mL of buffer per 10^8^ cells and tested against the human SMN standard protein at 0.063 to 4 ng/mL for the ProteinTech antibody 11708-1-AP and at 0.25–16 ng/mL for the SC-15320 antibody (Table S2).

### Validity of SMN ELISA signal detection

Lysates from GM03813c ells treated with siRNA against SMN transcript were assessed in the ELISA to confirm that the signals being detected in the ELISA are specific to SMN protein. GM03813 cells were cultured, lysed and processed as described in the ELISA proteosome inhibitor experiment with the exception that the cells were treated for 48 h with SMN siRNA at 10 µM and 50 µM (#L-011108-00-0005 from Dharmacon, Lafayette, CO), negative control (AM4635, Ambon, Austin, TX) or transfection reagent alone (Mock). The siRNA transfection mix was generated performed according to manufacturer instructions and allowed to form in serum-free media with interferrin for 10 minutes at room temperature. The siRNA/interferin complexes were added to 96-well plates (20 µl) and 7500 GM03813 cells were subsequently added to each well (180 µl). After 48 h incubating at 37°C, cells were harvested and lysed in preparation for analysis by ELISA and by Western blot. For the Western blots, cells from each well were lysed in 5 uL of Laemmli buffer, with 20 uL loaded into the gel. The blot SMN was detected using a mouse monoclonal BD antibody was used at 1∶1000 (#610646, San Diego, CA) with an Alexa Fluor680 goat anti-mouse IgG antibody (#A-21057, Invitrogen) as the secondary antibody. For the loading control a rabbit polyclonal GAPDH Santa Cruz Biotechnology antibody (#SC-25778) was used at 1∶1000, with a IRdye800 conjugated goat-anti-rabbit IgG at 1∶10000 as a secondary antibody (#611-132-122, Rockland Immunochemicals, Gilbertsville, PA) as a control. Blots were read on an Odyssey LI-COR system (Lincoln, Nebraska) in the 700 nm and 800 nm wavelength channels.

### ELISA protocol

Recombinant human SMN1 was generated from full-length cDNA expressed in bacterial expression vectors and purified for use as a standard in the ELISA. The mouse monoclonal capture antibody Sigma anti-SMN clone 2B1 (#S2944, St. Louis, MO) was coated using 100 uL onto Costar Stripwell (#92592, Lowell, MA) at 3.5 µg/mL. After overnight incubation at room temperature, the plate was blocked for 5 hours with 1% BSA in PBS. Cell lysate samples and recombinant hSMN or HeLa cell lysates (generated from lysis in ER4 buffer) standards were loaded at 100 µL per well. Standards were diluted in 2-fold dilutions or from 50–3200 pg/mL. Samples were incubated for one hour at room temperature, washed and then incubated with a rabbit polyclonal detection antibody raised against the product of a recombinant SMN2 gene from Proteintech Group (#11708-AP-1, Chicago, IL) at 2 µg/mL for one hour at room temperature. After washing, a peroxidase conjugated goat anti-rabbit IgG from Jackson Immunoresearch (#111-035-144, West Grove, PA) was applied at 50 ng/mL to the plate and incubated for 30 minutes at room temperature. After washing, plates were developed with TMB substrate for 30 minutes incubation at room temperature and the reaction stopped with 1 N HCl. Plates were then read on a spectrophotometer at 450 nm. Plates were sealed and gently shaken during all incubations, dilutions of sample and standard were made using assay buffer (1% BSA, 0.1% Tween-20 in PBS). The ELISA is available as a kit from Enzo Life Sciences (#ADI-900-209 Farmingdale, NY) and sold for research use only under license from INSERM (Paris, France).

### Cell lysis

A cell count with a hemocytometer was always performed immediately prior to lysis for accurate count of viable cells, which was used for determining volume of cell lysis buffer. Lysis buffer ER4 containing 300 mM NaCl, 10% glycerol, 3 mM EDTA, 1 mM MgCl_2_, 20 mM β-glycerophosphate, 25 mM NaF and 1% Triton X-100 and 0.1% Kathon was used along with protease inhibitors PIC8340 (Sigma #P8340) and phenylmethylsulphonyl fluoride (Sigma #P7626). PBMCs were thawed in a 37°C water bath and resuspended in ER4 with inhibitors at 10^8^ cells per milliliter. The cell suspension was gently vortexed and placed on ice for 30 minutes. The cell lysate was transferred to a 1.5 mL centrifuge tube and was clarified by centrifugation for 10 minutes at 14000RCF, 4°C. The supernatant was transferred to a clean vial and either assayed immediately or stored at −70°C until use. HeLa lysates are prepared in a similar manner with the exception that cells were lysed at a ratio of 1 mL per 10^6^ cells.

### ELISA and PBMC validation experiments

For the dilutional linearity experiment, six lots of control PBMCs were purchased from AllCells for further assay testing. After thawing and lysis, PBMCs were serially diluted 4-fold and compared to hSMN standard curve for linearity from 1∶4 to 1∶32 dilutions. To test spike recovery, PBMC lysates were diluted 1∶2 and 1∶4, with hSMN spikes of 267, 667, 1667 pg/mL and assessed by ELISA. The recovery value was calculated by subtracting the background hSMN concentration of the unspiked matrix from the concentration value, and dividing by the spiked concentration and multiplying it by 100. To determine reproducibility, hSMN standard was analyzed in N = 4 experiments. Sensitivity was interpolated by two standard deviations above mean signal at background. For parallelism analysis of native versus recombinant SMN, dose-response curves were made with HeLa cell lysates and hSMN standards and assessed by ELISA at dilutions from 1∶4 to 1∶512. For freeze-thaw experiments tubes of cell lysates thawed 0, 1, 2, or 3 times were assessed in the ELISA.

### Human biofluid and tissue analysis

Various human fluids and tissues were acquired to determine whether the SMN ELISA could detect signals in other matrices. Panels of plasma, saliva, urine, buccal swabs and PBMCs from 6 normal donors were acquired for SMN protein analysis from Analytical Biological Services (Wilmington, DE). Plasma, saliva and urine samples were thawed, gently mixed and transferred to 1.5 mL tubes for centrifugation at 1200 rpm for 10 minutes at 4°C. Plasma saliva and urine samples were serially diluted from 1∶2 to 1∶128. Buccal swab and PBMC samples were incubated with 200 to 400 µL of ER4 on ice for 30 minutes. Samples were vortexed twice during the incubation and then transferred to 1.5 mL tubes for processing as above. Buccal cells and PBMCs were serially diluted at 1∶2 to 1∶256 and 1∶4 to 1∶512 respectively, with SMN pg/mL results normalized to the mg/mL of total cellular protein levels as measured by protein quantitation assay. Human muscle samples (N = 4) were acquired from Asterand (Detroit, MI) and analyzed in the SMN ELISA and also in a desmin ELISA as a muscle-specific control from USCN Life Science (#E90373Hu, Burlington, NC). There were 4 muscle samples collected post-mortem from donors aged 37–97 years that died of coronary heart disease or accidental head trauma. Samples were homogenized using a polytron at medium speed for 2 to 3 pulses of 5 seconds each on ice in a 1∶5 ER4 dilution. The resulting lysates were tested in the SMN ELISA as described above while the desmin ELISA was tested as per manufacturer instructions.

### SMN measurement in a drug-treated SMA fibroblast cell line

Type I SMA fibroblast samples were acquired from Coriell (line #GM03813, Camden, NJ) and were thawed and incubated in in DMEM-10% FBS for 3 days. Cells were trypsinized, counted and resuspend to 25000 cells/ml in DMEM-10%FBS. Cell suspensions were plated at 5000 cells per well in a 96 well microtiter plate and incubated for 3 to 5 h. Proteosome inhibitors MG132 (0.001 to 1 µM) and bortezomib (0.001 to 100 nM) were prepared in 7-point dilution curve in DMSO. For each drug 1 µL of compound solution was added to each well and incubated for 24 h in a cell culture incubator (37°C, 5% CO_2_, 100% relative humidity). At the time of SMN analysis, supernatant was removed from the cells and 100 µL of the ER4 extraction buffer added per well. After shaking for 1 h at room temperature, 100 µL of cell lysate was transferred to appropriate wells in the SMN ELISA plate and processed as per described above. SMN signal was calculated as a fold over the signal detected in DMSO-treated negative control cells. Viability for cells in parallel treatment wells was assessed using a CellTitre-Glo assay from Promega (#7571, Madison, WI) according to manufacturer instructions.

### Comparison of human and mouse SMN reactivity in ELISA

Recombinant human SMN and mouse Smn standards were compared side-by-side for reactance at ranges of 50 to 3200 pg/mL and 390 to 50000 pg/mL respectively and developed as described above. General reactivity of mouse Smn was estimated as a percentage of the human SMN OD value curve.

### SMN protein detection in PBMCs across species

Whole blood from a non-human primate (Cynomologous macaque) was acquired for SMN ELISA analysis from BioQuant (San Diego, CA). Briefly, the monkey blood sample was diluted 1∶2 with room temperature PBS. Six milliliters of diluted sample was then layered onto 3 ml of Lymphoprep solution (#1114544, Axis-Shield, Oslo, Norway) in separate 15 ml centrifuge tubes. The samples were then centrifuged at 2500 rpm for 20 minutes at room temperature. The resultant upper layer was then harvested and cells pelleted by centrifugation at 1500 rpm for 10 minutes. The cell pellet was then suspended in 2 ml of erythrocyte lysis (EL) buffer (#00-4333-57, eBioscience, San Diego, CA) and incubated for 5 minutes at room temperature prior to centrifugation. Cells were washed with PBS to remove the EL buffer and resuspended in PBS and enumerated as previously described. Finally PBMC's were pelleted and resuspended in 200 µl of ER4 lysis buffer. Non-human primate PBMC lysates were run alongside lysates from normal human PBMCs (from AllCells) and mouse PBMCs harvested from 8 week old FVBn animals at PharmOptima. Samples were thawed and lysed at dilutions ranging from 1∶4 to 1∶32 in ER4 buffer at a ratio of 1 mL/10^7^ cells.

### Mouse tissue analysis

SMA ‘Δ7’ model mice (Smn^−/−^; hSMN2^+/+^; hSMN2Δ7^+/+^, Jackson Laboratory strain #005025) of homozygous, heterozygous and wild-type genotypes (N = 6 per genotype) were sacrificed at P3, P9, and P14 for tissue collection at PsychoGenics (Tarrytown, NY). Wild-type FVB mice were provided by Charles River Laboratories (Wilmington, MA) and sacrificed for collection at PharmOptima (Portage, MI). All animals had ad libitum access to food and water, were maintained in a 12∶12 hour light-dark cycle and were managed under protocols to promote ethical use by each facility's Institutional Animal Care and Use Committee (IACUC). SMA Δ7 mice were anesthetized with an intraperitoneal dose of 50–90 mg/kg pentobarbital. Blood was collected via cardiac puncture and stored at 4°C in EDTA tubes until centrifugation at 2000G for 15 minutes. Plasma and blood pellets were then stored at −80°C. Hemisected brains, whole spinal cord, and left and right gastrocnemius and soleus muscles were also collected and stored at −80°C. Following overnight shipment on dry ice, the mouse tissues were returned to −80°C storage at PharmOptima.

Adult wildtype FVB mice (N = 3/gender, 14 weeks old) were anesthetized in similar fashion, with blood kept on wet ice until processing to isolate peripheral blood mononuclear cells (PBMC). Tissues collected included hemisected brain, whole spinal cord, gastrocnemius muscle, liver, heart, abdominal adipose tissue, ear pinnae, and 2 mm punches from the ear pinnae. Following tissue collection, whole blood was diluted 1∶1 with 0.1 M PBS. Each diluted sample was layered onto 3 mL Lymphoprep solution. Samples were centrifuged at 800G for 30 minutes at room temperature (brake off). The white band at the interface with the gradient solution was transferred to a clean 15 mL tube. The cells were washed by adding approximately 13 mL PBS and centrifugation at 230G for 10 minutes at room temperature. The supernatant was discarded and the red blood cells lysed with 1 mL RBC lysis buffer (#00-4333-57, eBioscience, San Diego, CA). Approximately 13 mL of PBS was added to dilute the lysis buffer and then tubes were centrifuged at 230G for 10 minutes at room temperature. The supernatant was discarded and pellets resuspended by addition of 1 mL PBS. The total number of cells in all 4 hemocytometer squares was counted by trypan blue exclusion. Using a 1∶2 dilution, the number of viable cells that excluded the dye and the number of dead cells that did not exclude the dye was recorded.

Tissues were homogenized on ice in 4 to 30 µL ER4 per mg of wet weight with a minimum volume of 200 µL using either a Precellys tissue homogenizer or a polytron. Precellys homogenization was done at 5500 rpm for three 30 s cycles. Polytron homogenization used 3 separate 5 second pulses on ice at a medium speed. Samples were cleared via centrifugation at 14000G for 10 minutes at 4°C and supernatant transferred to a clean tube for protein quantitation using a BioRad DC protein assay (#500-0112, Hercules, CA), or a Thermoscientific BCA assay (#23225, Hudson, NH). Homogenates were by diluted to 200 µg/mL total protein concentration in assay buffer with subsequent dilutions specific to each tissue and genotype. Tests for spike-recovery of hSMN protein and tissue levels of SMN across were conducted with wild-type brain, muscle and spinal cord, while the ER4 lysis buffer was chosen for subsequent analysis of mouse tissues.

### CNS and motor circuit analysis of SMN protein

SMN was measured in wild-type mouse hemibrain, quadriceps muscle, spinal cord, and sciatic nerve. In the other mouse hemibrain, the cerebellum, cortex, hippocampus, olfactory bulb, striatum and thalamus was also dissected and processed for analysis. Due to the small mass of some tissues, they were homogenized by the Precellys system (Gaithersberg, MD). Tissues were weighed and 4 uL of ER4 lysis buffer was added for each mg of tissue. Samples were homogenized at 4°C at 5500 rpm at three cycles of 30 s per cycle and protein concentrations were assessed. ELISA analysis was done at dilutions ranging from 1∶5 to 1∶25.

### Comparison of SMN protein signal detection in mouse tissues

Delta7 mouse brains were analyzed in Western blot and the SMN ELISA. Tissues were harvested from N = 3 Delta7 KO and HET mice and homogenized in Laemmli buffer for Western blotting and the SMN ELISA as described in previous sections. Total protein for each sample was measured by the BCA assay and used to normalize the SMN signals and generate information for loading gels. Gels were loaded with 30 µg protein for the KO samples and 25 µg of protein for HET samples. ELISA results were expressed as SMN pg/mg total proteins and Western blot results were expressed as ratio of SMN to total protein.

### Statistical Methods

Analysis of statistical significance between SMN levels in mouse tissues was done by one-way ANOVA with Tukey's test or an unpaired t-test using Prism software by GraphPad (La Jolla, CA). P-values are indicated by asterisks or plus signs in the following manner: p<0.001 by ***, p<0.01 by ** and p<0.05 by *. In figures error bars depicted are standard deviations except where specified otherwise.

## Supporting Information

Figure S1 **Evaluation of coating buffers for SMN signal optimization.** Three coating buffers were tested against 2–8 µg/mL of human SMN recombinant protein for best signal. Coating buffers included 10 mM PO_4_ 15 mM NaCl pH 7.2, 10 mM PO_4_ pH 8.0, and 200 mM Carbonate-Bicarbonate pH 9.4. Error bars represent standard deviations.
(TIF)

Figure S2 **Titration of capture antibody in the SMN ELISA.** A titration experiment with capture antibody 2B1 was performed at 0, 0.5, and 16 µg/mL using 10 mM PO_4_ 15 mM NaCl pH 7.2 for the coating buffer. 3.5 µg/mL was selected as the coating concentration following 4-parameter analysis. Error bars represent standard deviations.
(TIF)

Figure S3 **Titration of detection antibody in the SMN ELISA.** Recombinant SMN protein was tested at 0, 0.5, and 16 ng/mLwith 62.5 to 2500 µg/mL of the Santa Cruz SMN detection antibody to evaluate the antibody concentration signal to noise values. Following this analysis 1.5 µg/mL was used for the detection antibody concentration.
(TIF)

Figure S4 **Comparability of SMN protein signal in mouse tissues with ELISA and Western blots.** Brain tissue from postnatal day 9 KO and postnatal day 50 HET Delta7 mice were homogenized and analyzed side-by-side in A: the SMN ELISA and B: Western blot. C: The image of the Western blot visually corroborates the results with the ELISA. Error bars represent standard deviations. P-values are indicated by asterisks or plus signs in the following manner: p<0.01 by ** and p<0.05 by *.
(TIF)

Table S1 Summary of reagents tested for SMN ELISA signal interference. Reagents were tested at a range of four concentrations in assay buffer (100 mM PO_4_, 150 mM NaCl, 1%BSA 0.1%Tween-20) and assay buffer with 16 ng/mL human SMN recombinant protein standard. Significant interference was observed with SDS and Sodium deoxycholate, as all concentrations tested caused 50% or greater reduction in SMN protein signal detection.
(DOCX)

Table S2 Comparison of detection antibody reactivity to SMN protein. Detection antibodies were tested with capture antibody 2B1 coated at 3.5 ug/mL. Recombinant hSMN was prepared in a dilution series 0.0625–8 ng/mL, HeLa lysate was prepared in 100 mM Tris, pH 7.5, 2.5% NP-40 extraction buffer and tested in a 1∶100 to 1∶625 dilution series.
(DOCX)

Table S3 Comparison of SMN extraction buffers. Extraction buffers were evaluated with human PBMCs using capture antibody 2B1 coated at 3.5 ug/mL. ER2 consisted of 100 mM Tris, pH 7.5, 2.5% NP-40, ER2+ contained 100 mM Tris, pH 7.5, 2.5% NP-40, 300 mM NaCl, 0.5% SDS, 25 mM NaF, 3 mM EDTA, 1 mM MgCl2, 20 mM β-Glycerophosphate, and ER4 contained 50 mM Tris, pH 7.5, 300 mM NaCl, 10% (w/v) glycerol, 3 mM EDTA, 1 mM MgCl2, 20 mM β-glycerophosphate, 25 mM NaF, 1% Triton X-100. CV = coefficient of variance. OD = optical density.
(DOCX)
