# Supplementary figures and images for: Utility of Survival Motor Neuron ELISA for Spinal Muscular Atrophy Clinical and Preclinical Analyses
Source: PLoS One. 2011 Aug 31;6(8):e24269. doi: 10.1371/journal.pone.0024269 (PMC3164180; doi:10.1371/journal.pone.0024269)

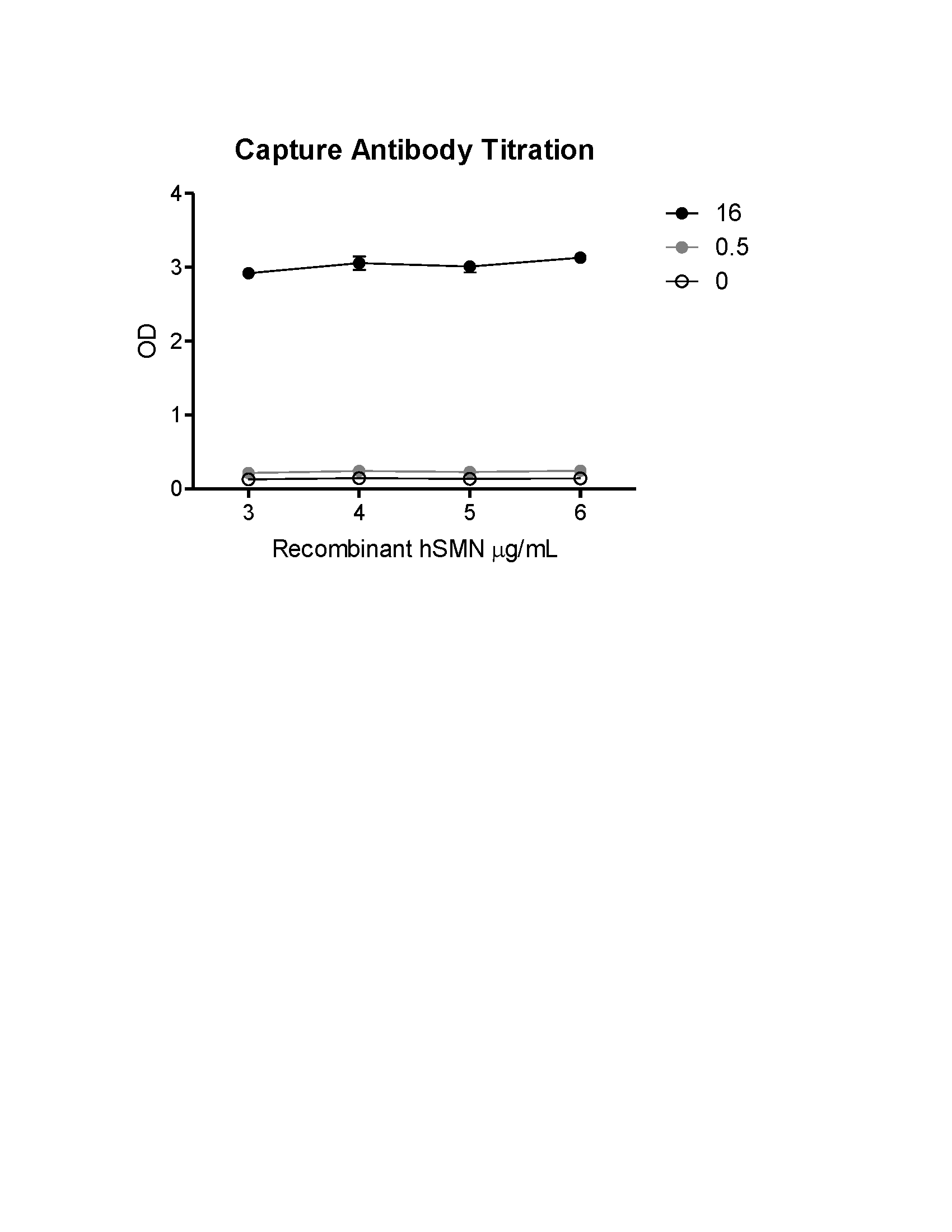

Supplement: Figure S2 — Titration of capture antibody in the SMN ELISA. A titration experiment with capture antibody 2B1 was performed at 0, 0.5, and 16 µg/mL using 10 mM PO4 15 mM NaCl pH 7.2 for the coating buffer. 3.5 µg/mL was selected as the coating concentration following 4-parameter analysis. Error bars represent standard deviations. (TIF) [file pone.0024269.s002.tif]

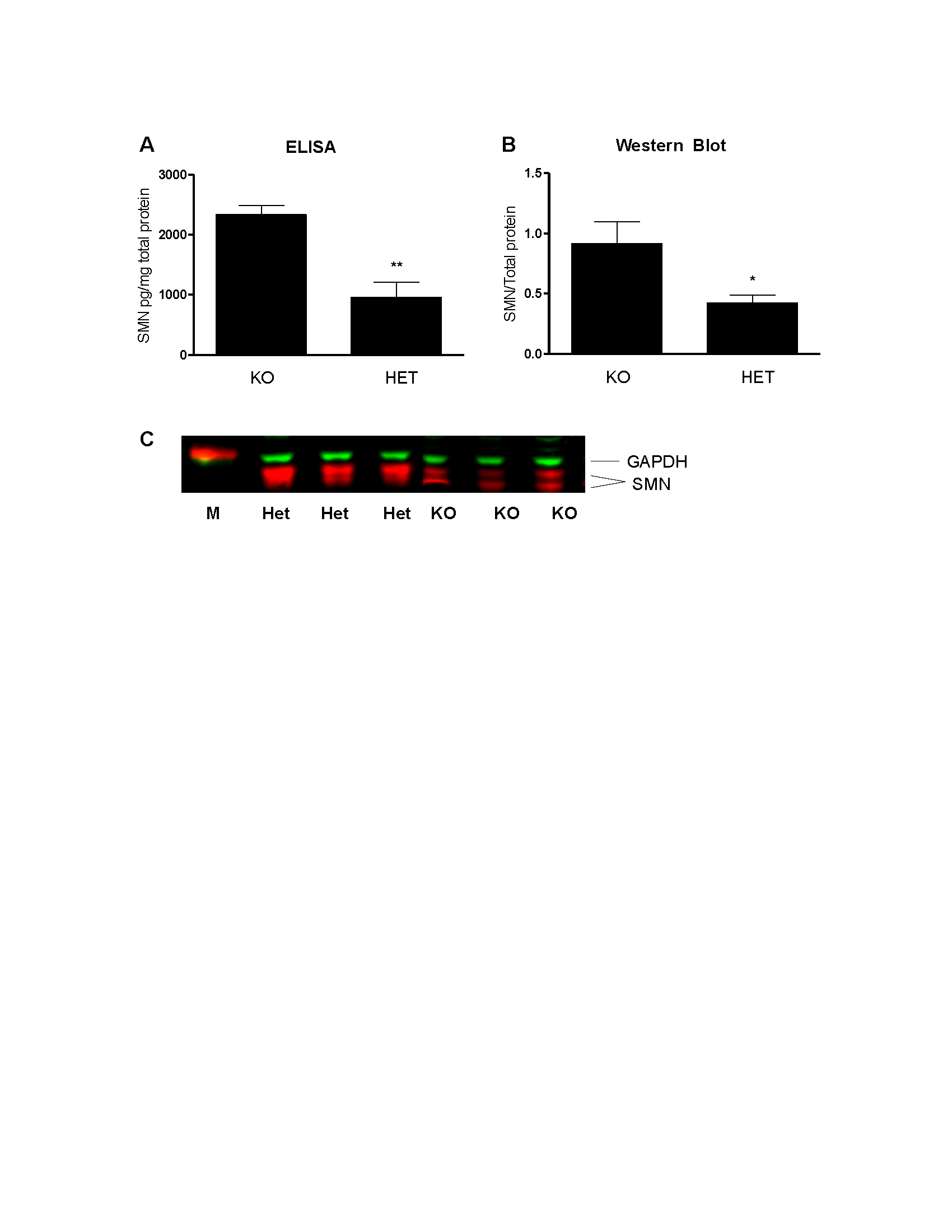

Supplement: Figure S4 — Comparability of SMN protein signal in mouse tissues with ELISA and Western blots. Brain tissue from postnatal day 9 KO and postnatal day 50 HET Delta7 mice were homogenized and analyzed side-by-side in A: the SMN ELISA and B: Western blot. C: The image of the Western blot visually corroborates the results with the ELISA. Error bars represent standard deviations. P-values are indicated by asterisks or plus signs in the following manner: p<0.01 by ** and p<0.05 by *. (TIF) [file pone.0024269.s004.tif]
