# Supplementary material for: Utility of Survival Motor Neuron ELISA for Spinal Muscular Atrophy Clinical and Preclinical Analyses
Source: PLoS One. 2011 Aug 31;6(8):e24269. doi: 10.1371/journal.pone.0024269 (PMC3164180; doi:10.1371/journal.pone.0024269)
Supplement: Table S1 — Summary of reagents tested for SMN ELISA signal interference. Reagents were tested at a range of four concentrations in assay buffer (100 mM PO4, 150 mM NaCl, 1%BSA 0.1%Tween-20) and assay buffer with 16 ng/mL human SMN recombinant protein standard. Significant interference was observed with SDS and Sodium deoxycholate, as all concentrations tested caused 50% or greater reduction in SMN protein signal detection. (DOCX) [file pone.0024269.s005.docx]

**Table S1. Summary of reagents tested for SMN ELISA signal interference**

| **Reagent** | **Low Concentration** | **High Concentration** | **ELISA Compatibility** |
| --- | --- | --- | --- |
| EDTA | 1mM | 8mM | No interference at concentrations tested |
| EGTA | 1mM | 8mM | **20% interference at 8mM** |
| NP-40 | 2% | 16% | **<8%** |
| PIC8340 | 0.5μL/mL | 4μL/mL | No interference at concentrations tested |
| PMSF | 1mM | 8mM | No interference at concentrations tested |
| SDS | 0.10% | 0.80% | **<0.05%** |
| Sodium deoxycholate | 0.06% | 0.5% | **<0.0625%** |
| Triton X-100 | 0.25% | 2.0% | No interference at concentrations tested |
| Zwittergent 3-14 | 0.006% | 0.05% | No interference at concentrations tested |
