# Supplementary material for: Utility of Survival Motor Neuron ELISA for Spinal Muscular Atrophy Clinical and Preclinical Analyses
Source: PLoS One. 2011 Aug 31;6(8):e24269. doi: 10.1371/journal.pone.0024269 (PMC3164180; doi:10.1371/journal.pone.0024269)
Supplement: Table S2 — Comparison of detection antibody reactivity to SMN protein. Detection antibodies were tested with capture antibody 2B1 coated at 3.5 ug/mL. Recombinant hSMN was prepared in a dilution series 0.0625–8 ng/mL, HeLa lysate was prepared in 100 mM Tris, pH 7.5, 2.5% NP-40 extraction buffer and tested in a 1∶100 to 1∶625 dilution series. (DOCX) [file pone.0024269.s006.docx]

**Table S2. Comparison of detection antibody reactivity to SMN protein**

| **Antibody** | **Immunogen** | **Recombinant hSMN reactivity** | **HeLa lysate reactivity** |
| --- | --- | --- | --- |
| Santa Cruz | 0.17ng/mL | 0.17ng/mL | 509ng/mL |
| Aviva | 0.2ng/mL | 0.2ng/mL | 489ng/mL |
| Novus | No detection | No detection | Not tested |
| ProteinTech | ~0.05ng/mL | ~0.05ng/mL | 483ng/mL |
