# Supplementary material for: Utility of Survival Motor Neuron ELISA for Spinal Muscular Atrophy Clinical and Preclinical Analyses
Source: PLoS One. 2011 Aug 31;6(8):e24269. doi: 10.1371/journal.pone.0024269 (PMC3164180; doi:10.1371/journal.pone.0024269)
Supplement: Table S3 — Comparison of SMN extraction buffers. Extraction buffers were evaluated with human PBMCs using capture antibody 2B1 coated at 3.5 ug/mL. ER2 consisted of 100 mM Tris, pH 7.5, 2.5% NP-40, ER2+ contained 100 mM Tris, pH 7.5, 2.5% NP-40, 300 mM NaCl, 0.5% SDS, 25 mM NaF, 3 mM EDTA, 1 mM MgCl2, 20 mM β-Glycerophosphate, and ER4 contained 50 mM Tris, pH 7.5, 300 mM NaCl, 10% (w/v) glycerol, 3 mM EDTA, 1 mM MgCl2, 20 mM β-glycerophosphate, 25 mM NaF, 1% Triton X-100. CV = coefficient of variance. OD = optical density. (DOCX) [file pone.0024269.s007.docx]

**Table S3. Comparison of SMN extraction buffers**

| **Antibody** | **Extraction buffer** | **Assigned SMN pg/10^6^ PBMCs** | **OD range** | **CV% range** |
| --- | --- | --- | --- | --- |
| Santa Cruz | ER4 | 87.7 | 0.26-1.48 | 0.5-4 |
|  | ER2+ | 26.8 | 0.26-1.31 | 4-12 |
|  | ER2 | 82.9 | 0.30-0.53 | 1-7 |
| ProteinTech | ER4 | 81.4 | 0.29-3.4 | 1-8 |
|  | ER2+ | 21.4 | 0.31-3.5 | 6-16 |
|  | ER2 | 95 | 0.37-1.1 | 1-4 |
